# F-18 FDG PET/CT Imaging in Normal Variants, Pitfalls and Artifacts in the Abdomen and Pelvis

**DOI:** 10.3389/fnume.2021.826109

**Published:** 2022-01-17

**Authors:** Mboyo D. T. Vangu, Jaleelat I. Momodu

**Affiliations:** Department of Nuclear Medicine and Molecular Imaging, Charlotte Maxeke Johannesburg Academic Hospital, University of the Witwatersrand, Johannesburg, South Africa

**Keywords:** FDG, abdomen, pelvis, PET/CT, pitfalls, variants

## Abstract

Since its introduction into clinical practice, multimodality imaging has revolutionized diagnostic imaging for both oncologic and non-oncologic pathologies. ^18^F-fluorodeoxyglucose (^18^F-FDG) PET/CT imaging which takes advantage of increased anaerobic glycolysis that occurs in tumor cells (Warburg effect) has gained significant clinical relevance in the management of most, if not all oncologic conditions. Because FDG is taken by both normal and abnormal tissues, PET/CT imaging may demonstrate several normal variants and imaging pitfalls. These may ultimately impact disease detection and diagnostic accuracy. Imaging specialists (nuclear medicine physicians and radiologists) must demonstrate a thorough understanding of normal and physiologic variants in the distribution of ^18^F-FDG; including potential imaging pitfalls and technical artifacts to minimize misinterpretation of images. The normal physiologic course of ^18^F-FDG results in a variable degree of uptake in the stomach, liver, spleen, small and large bowel. Urinary excretion results in renal, ureteric, and urinary bladder uptake. Technical artifacts can occur due to motion, truncation as well as the effects of contrast agents and metallic hardware. Using pictorial illustrations, this paper aims to describe the variants of physiologic ^18^F-FDG uptake that may mimic pathology as well as potential benign conditions that may result in misinterpretation of PET/CT images in common oncologic conditions of the abdomen and pelvis.

## Introduction

The introduction of multimodality imaging into clinical practice has revolutionized diagnostic imaging for both oncologic and non-oncologic pathologies. In recent years, Positron Emission Tomography combined with Computed Tomography (PET/CT) has become an essential hybrid imaging modality in oncology; playing a critical role in the detection, staging, assessment of treatment response, restaging and prognosis of a wide range of malignancies (1).

PET/CT hybrid imaging has the advantage of providing data from both physiological and anatomical changes at the same time. However, many physiologic conditions, normal variants, and benign lesions within the pelvis and abdomen may cause uncertainty during image interpretation. Also, the process of fusion of CT with PET for anatomical localization and attenuation correction can result in imaging artifacts. Furthermore, low ^18^F-FDG uptake in certain tumors such as mucinous tumors and indolent lymphomas can present a diagnostic challenge due to false-negative imaging results.

Accurate reporting relies on a thorough understanding of normal and variant distribution of FDG, including potential technical artifacts and imaging pitfalls. Clinical history and examination are critical including information on previous surgery, fasting state, level of glycaemia, use of insulin and exposure to cold.

## Normal Distribution of FDG

FDG is a glucose analog thus its physiologic distribution reflects glucose utilization in normal body tissues and organs. This paper will limit the brief description of normal distribution and normal as well as its variants to the abdomen and pelvis.

The liver usually shows a homogenous uptake that is greater than the uptake in spleen. There is generally a mild FDG uptake in the gastroesophageal junction. Moderate diffuse uptake is common in the stomach. Large and small bowel uptake is variable but greater in the caecum and ascending colon. Anal uptake is seen because of local sphincter activation and action of aerobic bacteria (2).

Normal renal excretion of FDG leads to visualized activity in the kidneys, renal pelvis, and urinary bladder. In the ureters, there may be continuous or intermittent uptake, as determined by ureteric peristalsis.

FDG uptake in the reproductive tract varies with the menstrual cycle- endometrial uptake is highest during the ovulatory and secretory phases. Ovarian uptake is usually seen in midcycle.

Normal symmetric uptake in the testes reduces with increasing age.

## Normal Physiologic Variants

**Figure 1:**
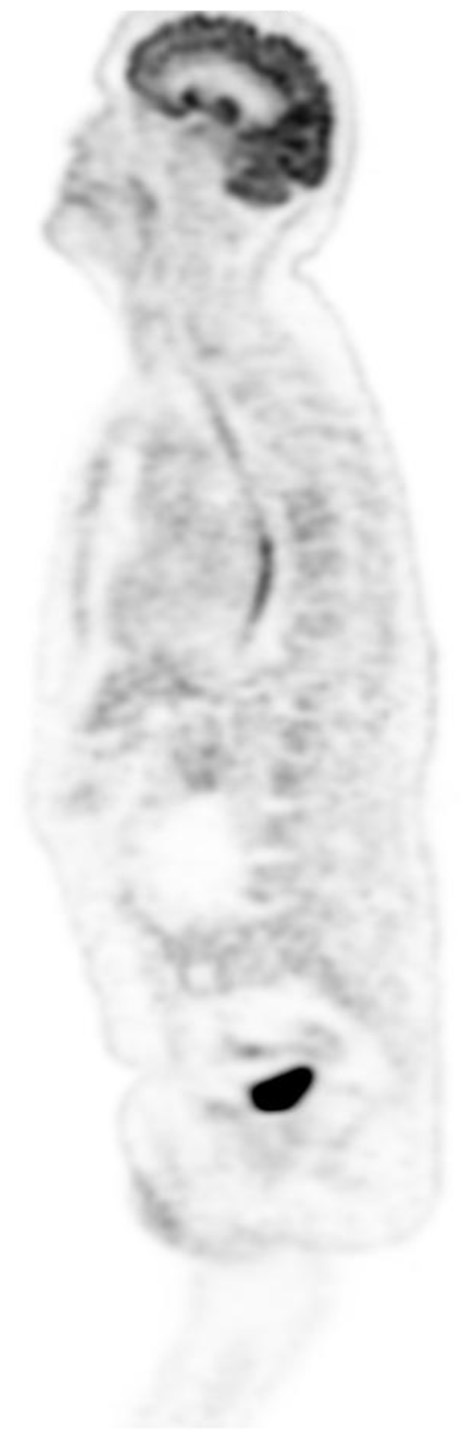
Severe reflux. FDG PET sagittal MIP image demonstrates diffuse uptake in esophagus and much increased in the lower third, in a 65-year-old male patient referred for therapy evaluation and he is known with gastro-esophageal reflux.

Normal focal low to moderate uptake can be seen at the gastroesophageal junction. However, an SUVmax >4 in a focal uptake is worrisome for pathology and warrants further work-up (3). Benign diseases can also demonstrate FDG uptake mimicking malignant pathology. In gastro-esophageal reflux disease, FDG uptake is typically at the distal third of the esophagus (Figure 1). Physiologic diffuse FDG uptake occurs in the gastric musculature, ranging from intense in the fundus to moderate and mild distally (3). The mechanism of this physiological pattern is unclear. However, it has been postulated to be related to higher FDG uptake in gastric parietal cells which are more numerous in the proximal part of the stomach (4). Physiologic gastric FDG uptake can also be related to normal lymphoid tissues.

**Figure 2:**
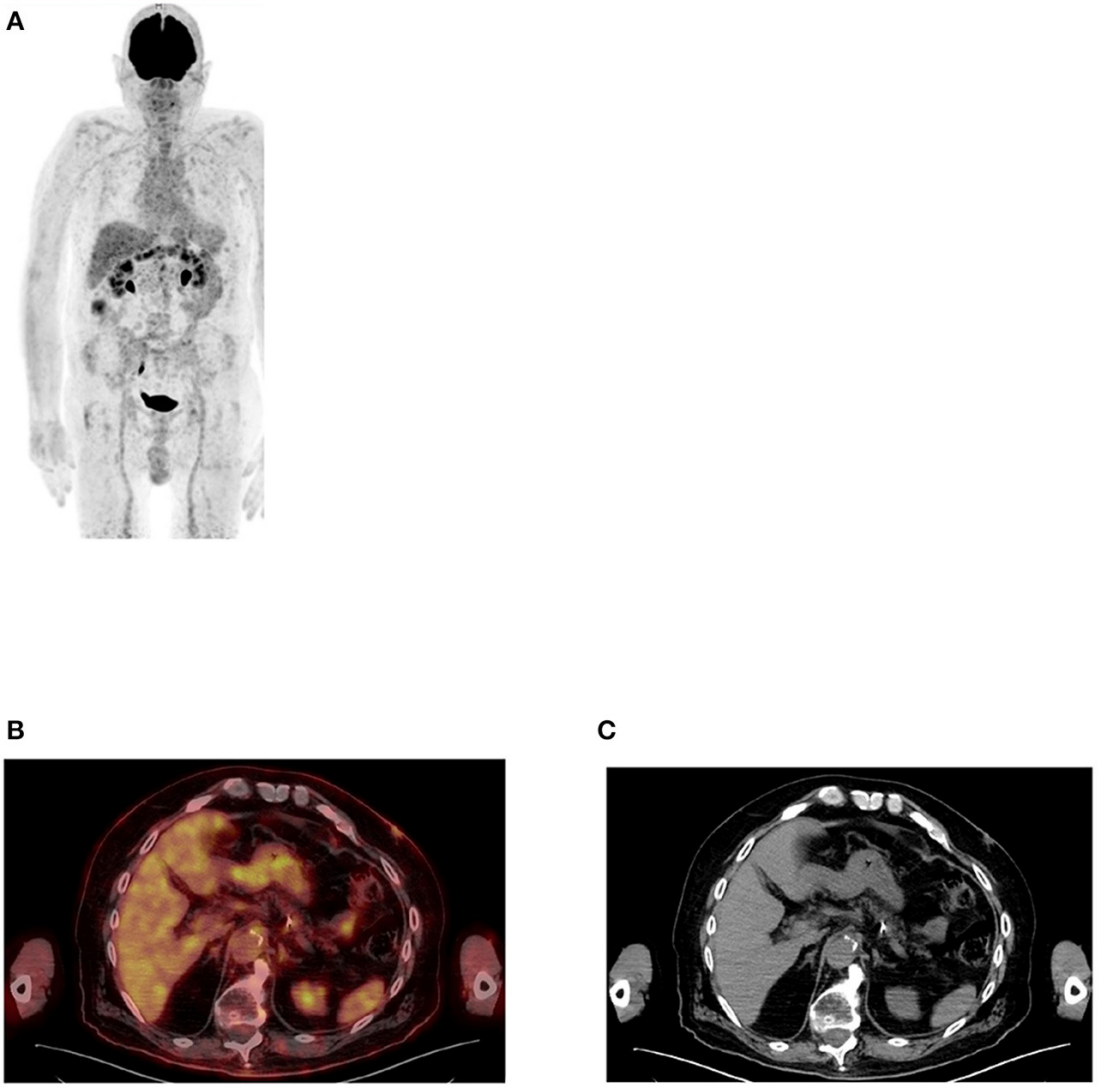
FDG PET MIP image **(A)** showing uptake in large, mainly in the transverse colon while the small bowel is almost not seen. This is a common finding in most patients who undergo PET/CT imaging. Axial PET/CT image **(B)** enhanced the describe finding in the large bowel with less uptake in small bowel that may be identified in the corresponding anatomical CT **(C)**.

**Figure 3:**
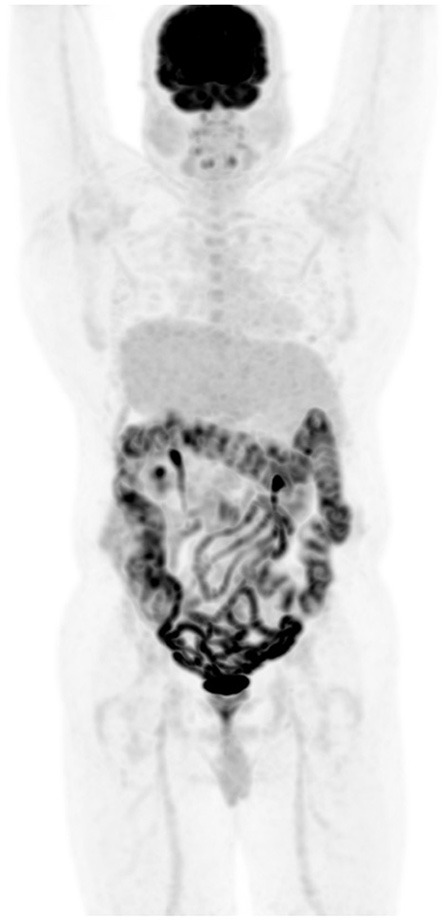
Metformin and bowel uptake. FDG PET MIP image demonstrates diffuse increased bowel uptake, in a diabetic patient who stopped medication 24 h before the scheduled PET/CT study visualization of large bowel with impressive details of the small bowel. Possibly, the increased sensitivity of the digital PET machine may also play a role of the visualized small bowel.

Diffuse FDG uptake in the bowel is frequently seen because of several factors that include the following: intestinal peristaltic activity, concentration in lymphoid tissue, mucosal activity and the presence of intestinal bacteria (3, 5). The large bowel activity is usually greater than that of the small bowel (Figure 2). Prominent increased large bowel uptake is generally seen in patients treated with metformin and may be diffuse, multifocal, or nodular (6). While small bowel does not usually show prominent uptake. However, due to increased sensitivity in newer digital PET/CT machines, FDG uptake in small bowel may show similar details to the large bowel (Figure 3). Requesting patients to temporarily discontinue treatment for about 2 days prior to study may decrease intestinal uptake while measures like bowel lavages are not effective (6).

**Figure 4:**
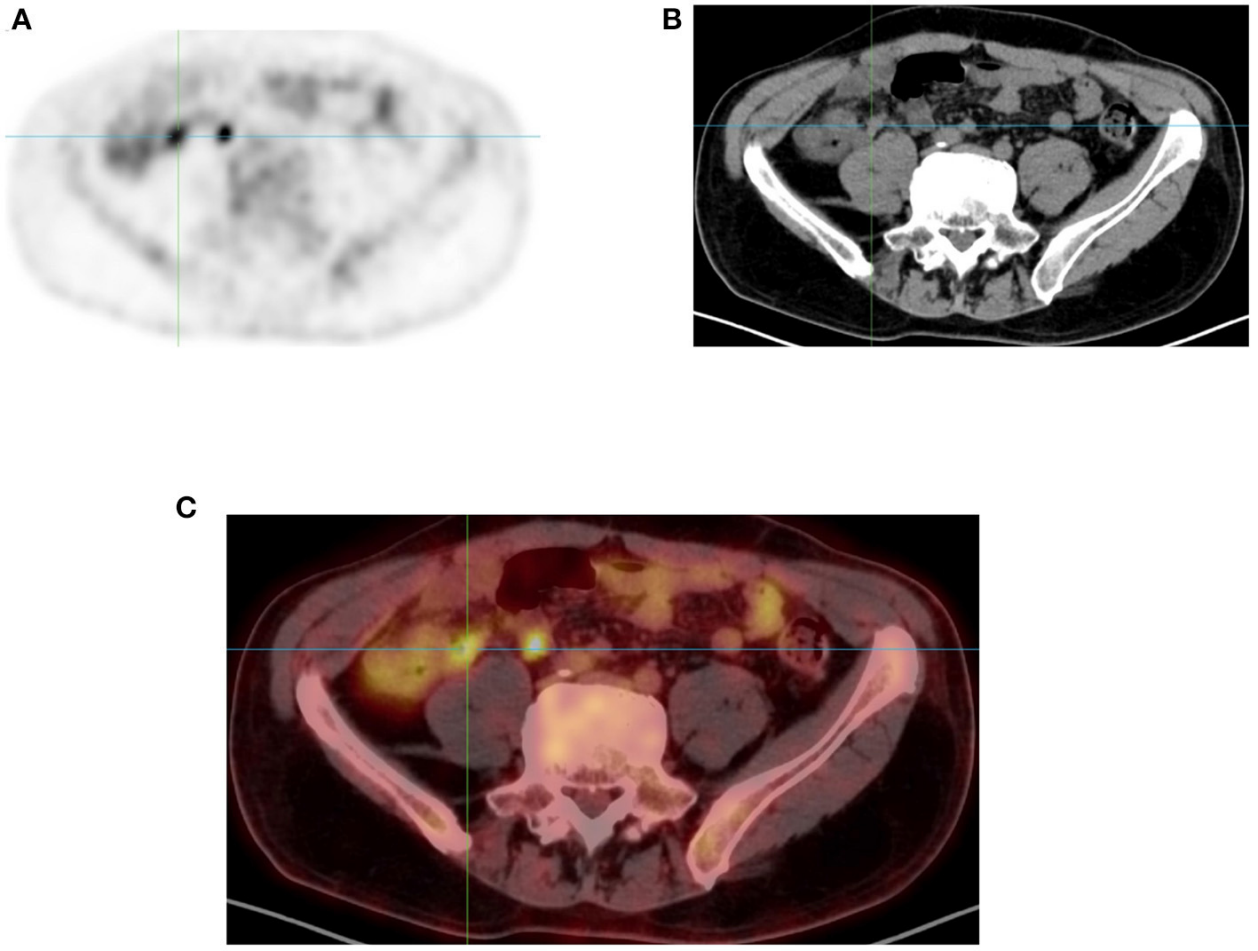
Appendix uptake. FDG PET Axial image **(A)** showing linear increased uptake in right iliac fossa that corresponds to appendix as correlated in anatomical CT **(B)** and combined PET/CT **(C)** images of the same plane (see crosshair in each image).

Physiologic large bowel uptake is most prominent in the cecum, ascending colon and rectosigmoid colon where the typical pattern of uptake is segmental with a mild to moderate intensity (3, 4). A nodular pattern of uptake is more typical for the ascending colon (3). Normal appendix may also show FDG avidity that is difficult to separate from neoplastic disease (Figure 4).

Benign pathologies that demonstrate FDG uptake include inflammatory bowel disease, infection, radiation, or medication-induced enteritis. In these conditions, the pattern of FDG uptake is linear along the affected bowel segments (4). Focal uptake in the descending colon can be due to constipation. Sphincteric activity, lymphatic tissue and fecal microbes within the anal canal can result in variable physiologic FDG uptake (4). Splenunculi may be FDG Avid and consequently mistaken for peritoneal tumors. This can be confirmed by demonstrating uptake on heat damaged red blood cells (4).

FDG is excreted through the urinary pathway, therefore in individuals with hydronephrosis, more prominent and intense tracer concentration can be observed in the renal pelvis, ureters, and bladder. There is usually symmetric ureteric uptake due to urine outflow.

## Artifacts

Artifacts generally occur independently of the PET tracer used. They can result in an apparent increase or decrease in tracer accumulation in the areas of potential clinical interest. These artifacts may cause inaccurate spatial registration between PET and CT data (2).

**Figure 5:**
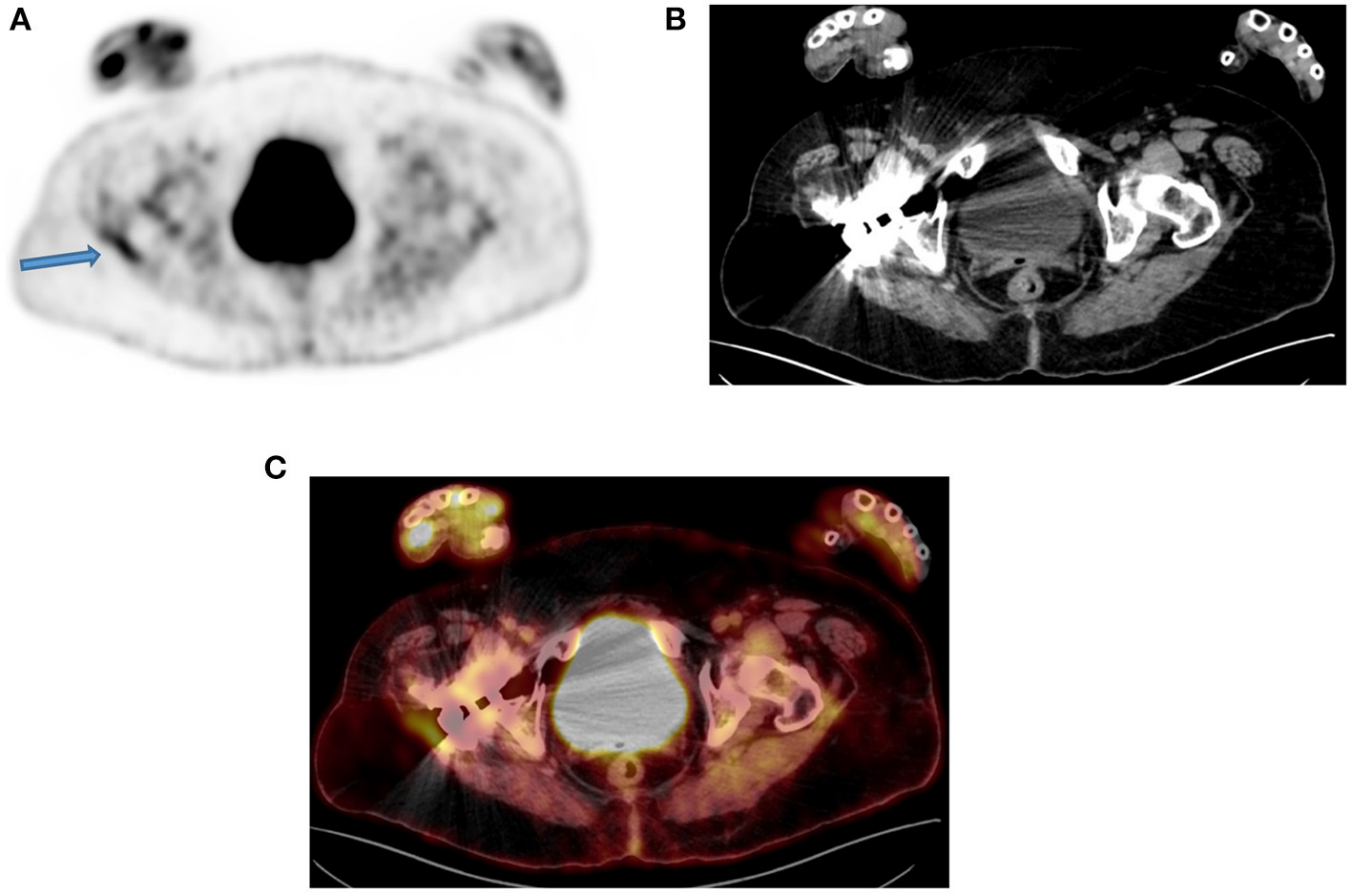
A 50-year-old man with a prosthesis in his right hip join. A PET attenuation-corrected image **(A)** shows FDG uptake in the lateral aspect of the prosthesis (solid arrow) l) caused by an artifact induced by attenuation correction. Clearly, no corresponding tissue is seen on the CT of the same region, despite the streaks effects from the metal implant **(B)** and confirmed in the fused PET/CT axial image **(C)**. When not sure on the reason of increased FDG uptake, interpreting physicians must assess the non-attenuation-corrected image of the same region to prevent misinterpretation.

**Figure 6:**
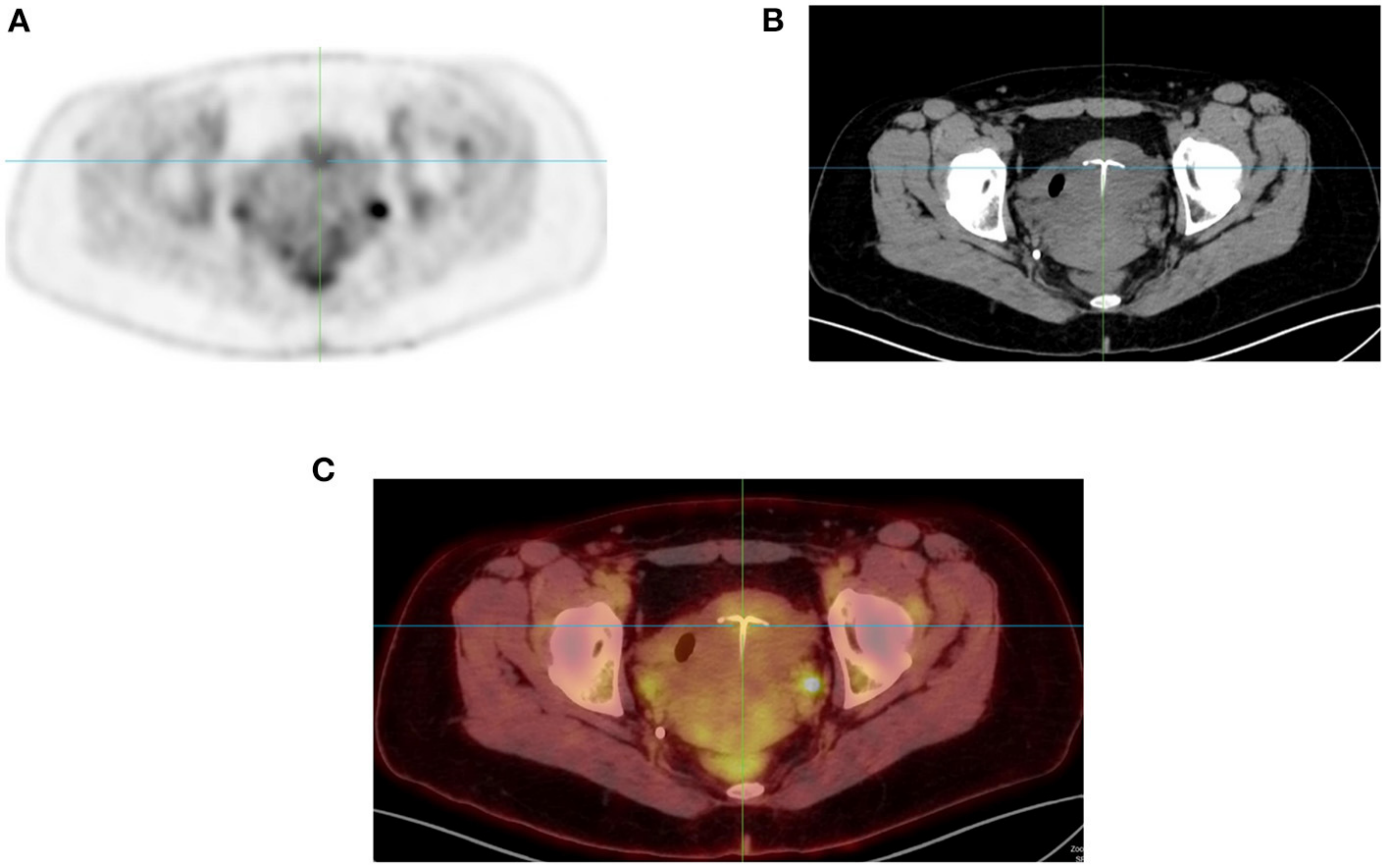
Uptake in intrauterine device (IUD). FDG PET MIP image in axial plane **(A)** showing focal uptake (Arrow) in anterior uterus. Corresponding CT **(B)** and combined PET/CT **(C)** confirms the uptake in the IUD.

**Figure 7:**
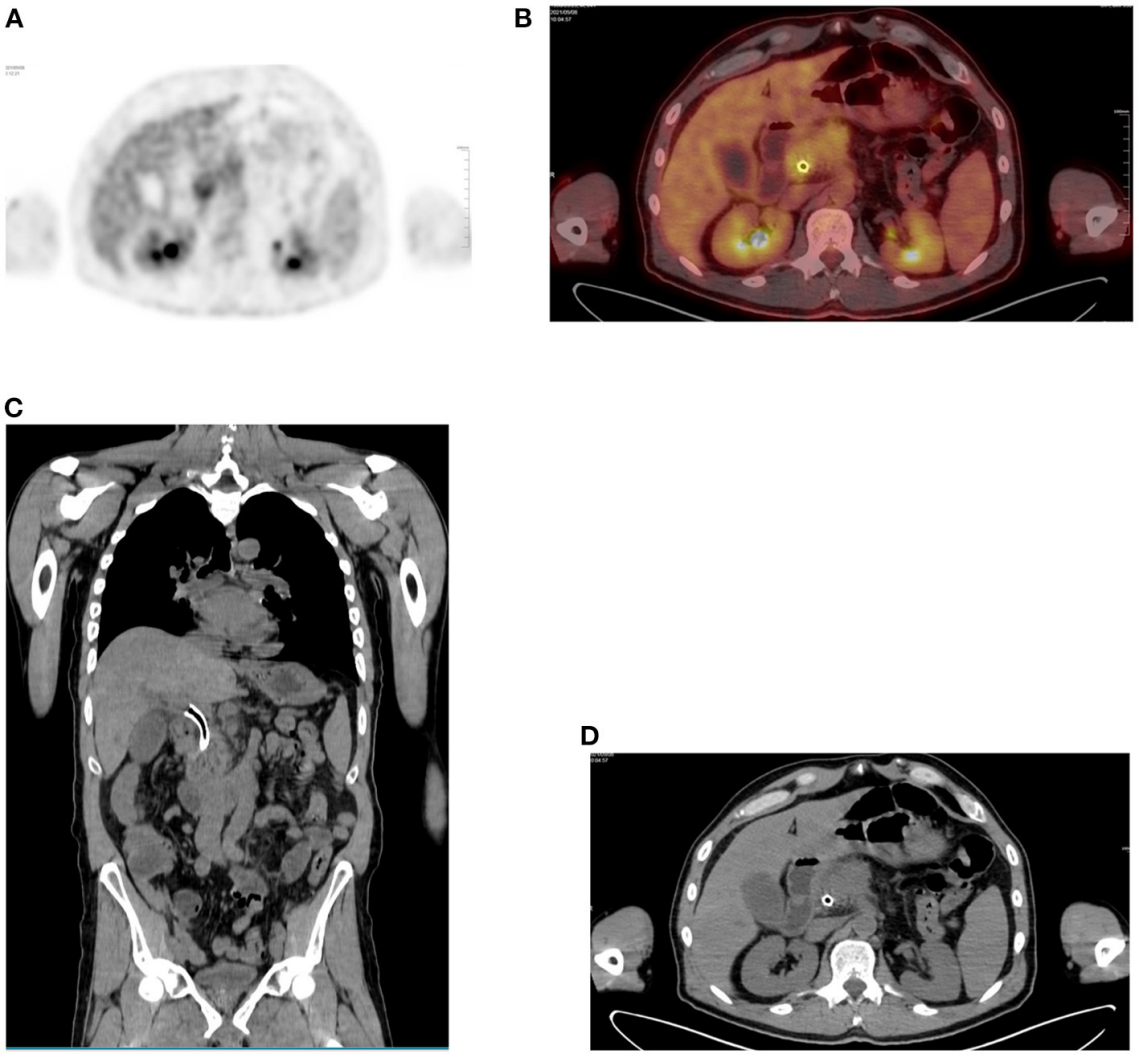
Focal uptake due to stent. **(A)** FDG PET Axial and **(B)** combined PET/CT with focal liver uptake in a 64-year-old male patient with pancreas adenocarcinoma and who underwent a placement of stent at CBD. Corresponding anatomical images alone, both coronal and axial planes **(C,D)** show the stent in place.

- **Motion Artifact (Voluntary, Involuntary) and Misregistration Can Artifactually Increase or Decrease Uptake:** Motion may be involuntary (cardiac contraction or respiratory movements) or voluntary such as gross patient movement during or in between CT and PET acquisitions (6). Motion artifacts typically manifest as blurring in PET images or as misalignment between PET and CT data sets which can lead to both spatial localization errors and incorrect attenuation correction leading to erroneously increased or decreased tracer accumulation in areas of clinical interest (2).
- **Oral Contrast:** Improves visualization of luminal, intramural, and extramural bowel disease by distending the bowel. Normal loops of bowel that have been distended with oral contrast typically exhibit low FDG uptake (5). The value of oral contrast is limited by attenuation artifacts produced during reconstruction of high density contrast in the bowel- due to inconsistencies in attenuation correction and failure to correctly scale the CT contrast agents to PET attenuation-correction maps (2, 5). This may result in foci of erroneously increased PET activity. These artifacts can be confirmed by reviewing the non-attenuation corrected (NAC) images which do not exhibit a high FDG uptake, confirming the attenuation artifact. Contrast material in the colon on CT may show increased uptake on AC image. However, on the NAC image, there is no or less activity thus indicating that the visualized area of increased activity is simply due to a reconstruction artifact. Low and medium- density oral contrast agents are available to overcome this limitation. However, most of patients in our department are given water as we seldom use oral contrast.
- **Attenuation Artifacts:** Like CT contrast agents, metal implants may not be correctly scaled for attenuation correction of PET data. Absorption of x-ray photons by the metal implant can result in streaking artifacts on CT images (2). Common areas that correspond to high-attenuation structures on CT include hip prostheses (Figures 5, 6), surgical clips, pacemakers and less often intrauterine contraceptive devices (Figure 7). CT attenuation in these instances may introduce attenuation artifacts due to overcorrection that may produce increased uptake capable to mimic or mask disease. Interpreting physicians should therefore look at both attenuation and NAC images to avoid mistakes.
- **Truncation Artifact:** In most available commercial PET/CT systems, the transaxial field of view of the CT component is smaller than that of the PET component. As a result, the anatomy covered in the PET images could be truncated in CT. This issue is more commonly see in large/obese patients (7).

## Pitfalls

PET/CT has become a valuable imaging modality in management of malignant diseases. As its usage is increasing, knowledge of potential pitfalls becomes crucial to prevent erroneous interpretations. This paper focuses on the abdomen and pelvis, therefore issues related to “brown fat” and muscular uptake are not discussed as they may be found in similar publications on melanoma, lymphomas and musculo-skeletal.

**Figure 8:**
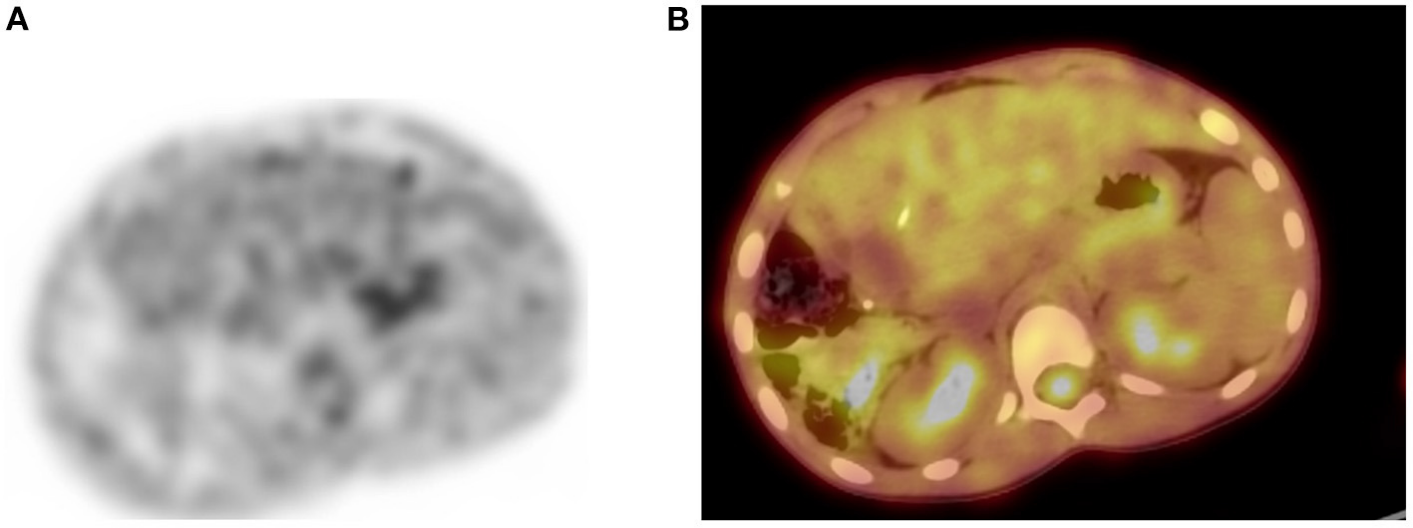
Diffuse gastric uptake. **(A)** FDG PET Axial and **(B)** combined PET/CT, showing diffuse uptake in inferior greater curvature of the stomach of a young patient who received a liver transplant. He continues to complain of abdominal pain following transplant procedure and all his inflammatory markers were raised. The visualized increased diffuse uptake was suggestive of gastritis and gastroscopy confirmed inflammation.

In the liver, changes related to either cirrhosis or hepatitis are readily seen on CT than PET/CT. However, interpretating physicians should be aware of relative increased FDG activity in the liver of patients with cirrhosis and chronic hepatitis that may impact the detection of hepatocellular carcinoma [HCC] (3). Patients with recent stent placement may show focal increased biliary tract FDG uptake (Figure 8) that may mimic nodal or liver metastasis as a false positive finding (3). Similarly, any surgical intervention may demonstrate increase uptake related to reactive inflammation and delaying the performance of PET/CT for at least is required (8).

Although hyperglycemia may affect almost all FDG studies, it has a more significant impact in imaging pancreatic carcinoma with a risk of cause a high rate of false negative findings. Caution must be observed in interpreting PET imaging in both diabetic and non-diabetic individuals with high levels of glucose (9, 10). This risk of false negative is common in tumors of early stages and increasing serum glucose while false positive may result from pancreatitis and benign pancreatic lesions (3). Biliary tract stent may demonstrate a focal uptake that is difficult to distinguish from a pancreatic lesion.

**Figure 9:**
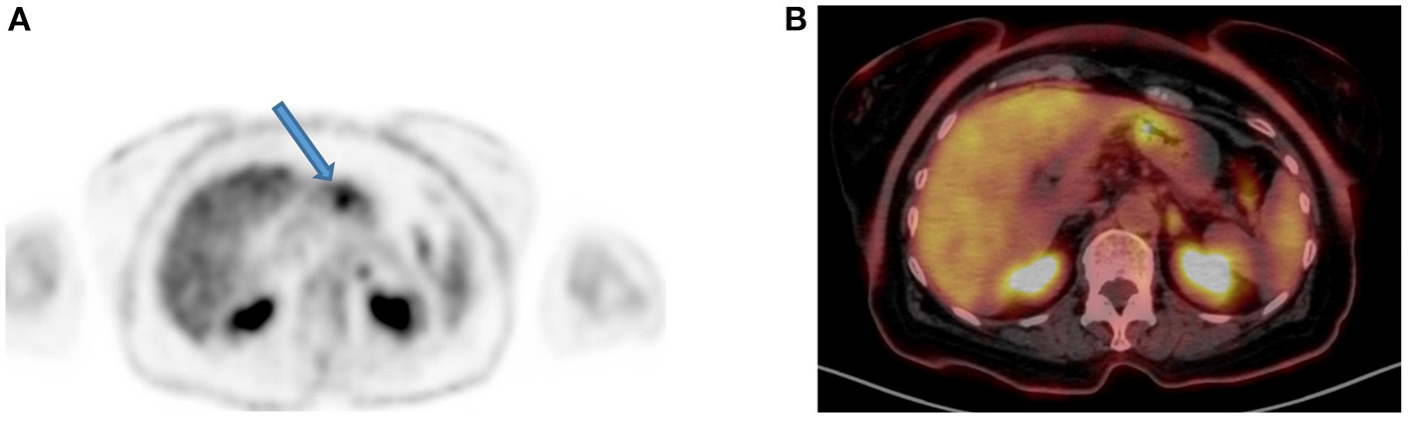
Focal gastric uptake. A 76-year-old female with newly diagnosed poorly differentiated gastric adenocarcinoma. **(A)** A focus FDG PET seen in Axial image (arrow) and **(B)** confirmed in the combined PET/CT. Focal uptake in gastric wall is typical for carcinoma and there was gastric outlet obstruction seen.

**Figure 10:**
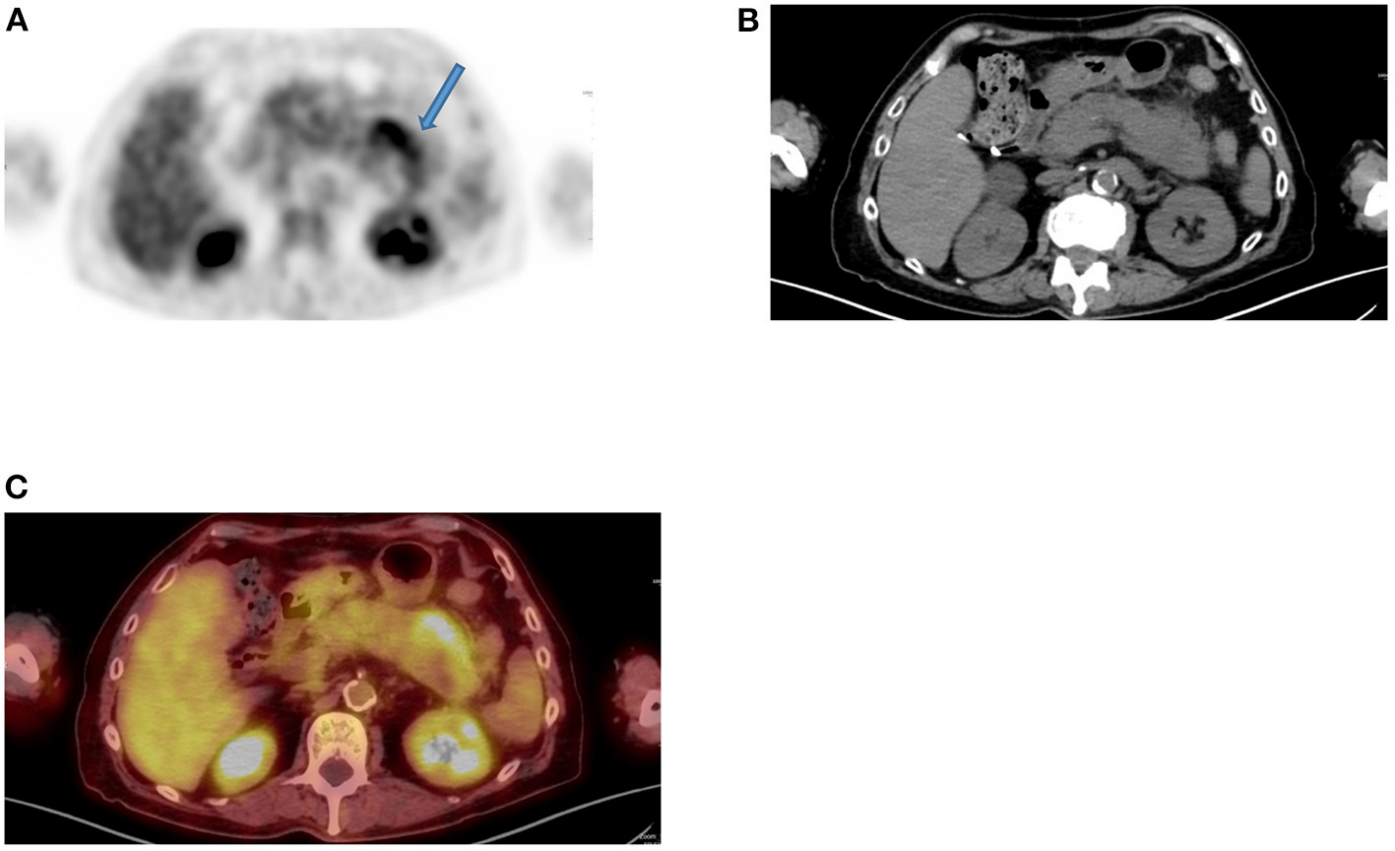
Inflammation uptake. FDG PET Axial image **(A)** demonstrates diffuse increased uptake in left central abdomen (arrow) that corresponds to bulky and edematous tail of pancreas **(B,C)**, in an 82-year-old male patient with previous history of rectal cancer and suspected liver metastases. The finding is consistent with chronic inflammatory process due to pancreatitis.

In gastric carcinoma, histology is key in the interpretation of FDG PET uptake. Generally, lower activity of FDG is linked to both signet ring cell and mucinous carcinoma. The decreased uptake seen in poorly differentiated disease is due to diffuse infiltration of the gastric wall while increased uptake in well-differentiated tumors is related to mass formation (3). Other causes of increased FDG activity in stomach are related to gastritis and lymphoma, both usually with diffuse uptake whereas gastric carcinoma typically has local uptake (Figures 9, 10). One suggested way to distinguish the high gastric activity of lymphoma from carcinoma is to look at the ratio of SUVmax to maximum wall thickness (11). Although perigastric nodes are included in dissection in individuals with advanced disease, it worth noting that they usually have uptake that is difficult to distinguish from uptake in adjacent tumor or physiologic gastric wall activity (3).

**Figure 11:**
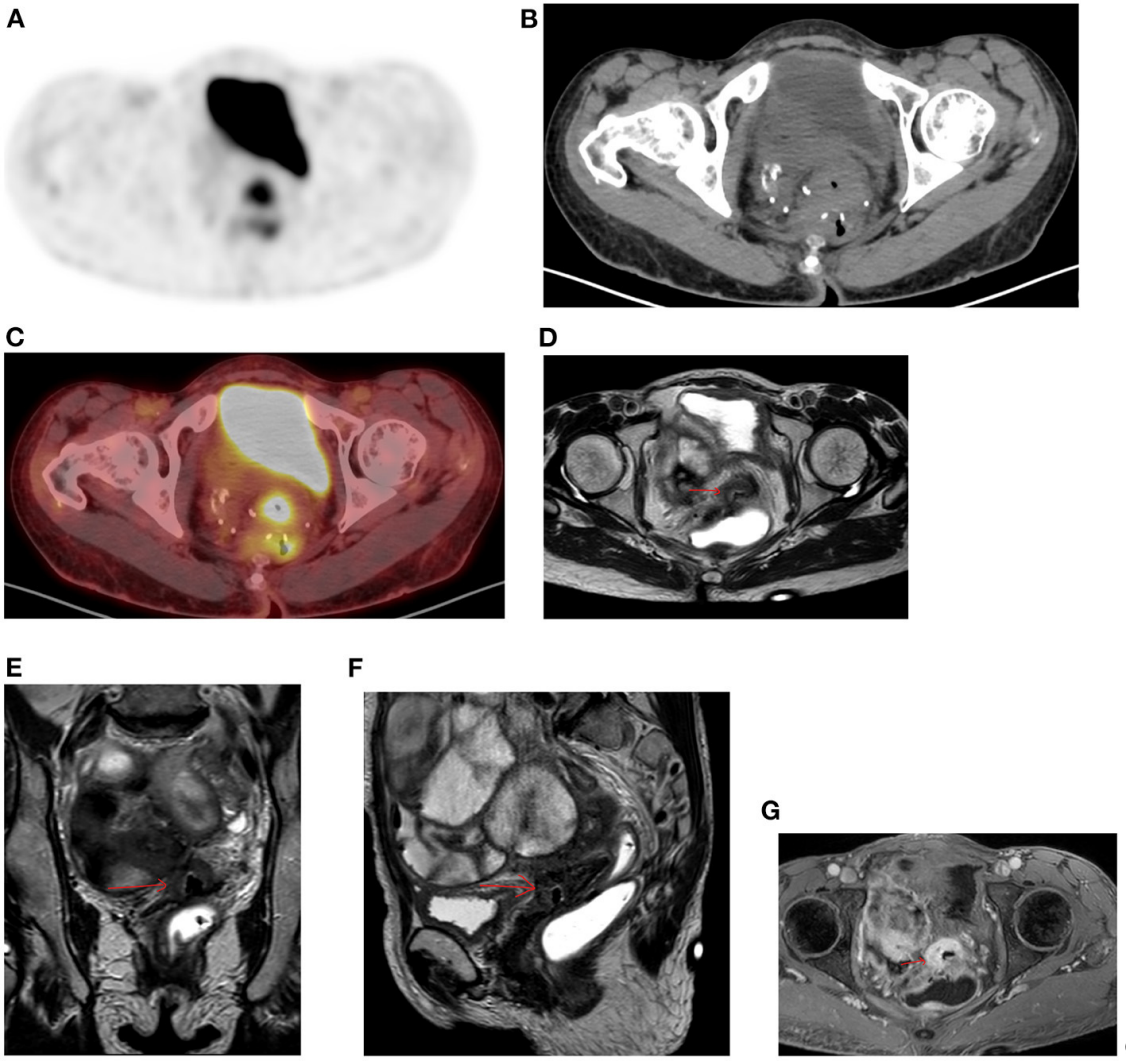
Diffuse uptake in pelvis. FDG PET Axial image **(A)** demonstrates diffuse increased uptake in central pelvis posterior to bladder that may be consider normal. The corresponding CT **(B)** showed a soft tissue mass with central air at the suture lines in a 56-year-old female patient with previous history of rectal cancer and had a complete response to chemotherapy. She was referred for restaging due to weight loss and the combined PET/CT Axial **(C)** image suspected a metastatic lesion despite diffuse uptake. MRI of pelvis was performed, and the Axial, coronal, and sagittal planes **(D–F)** confirmed the suspicious and showed overt enhancement of the soft tissue mass **(G)** that is consistent with metastasis.

**Figure 12:**
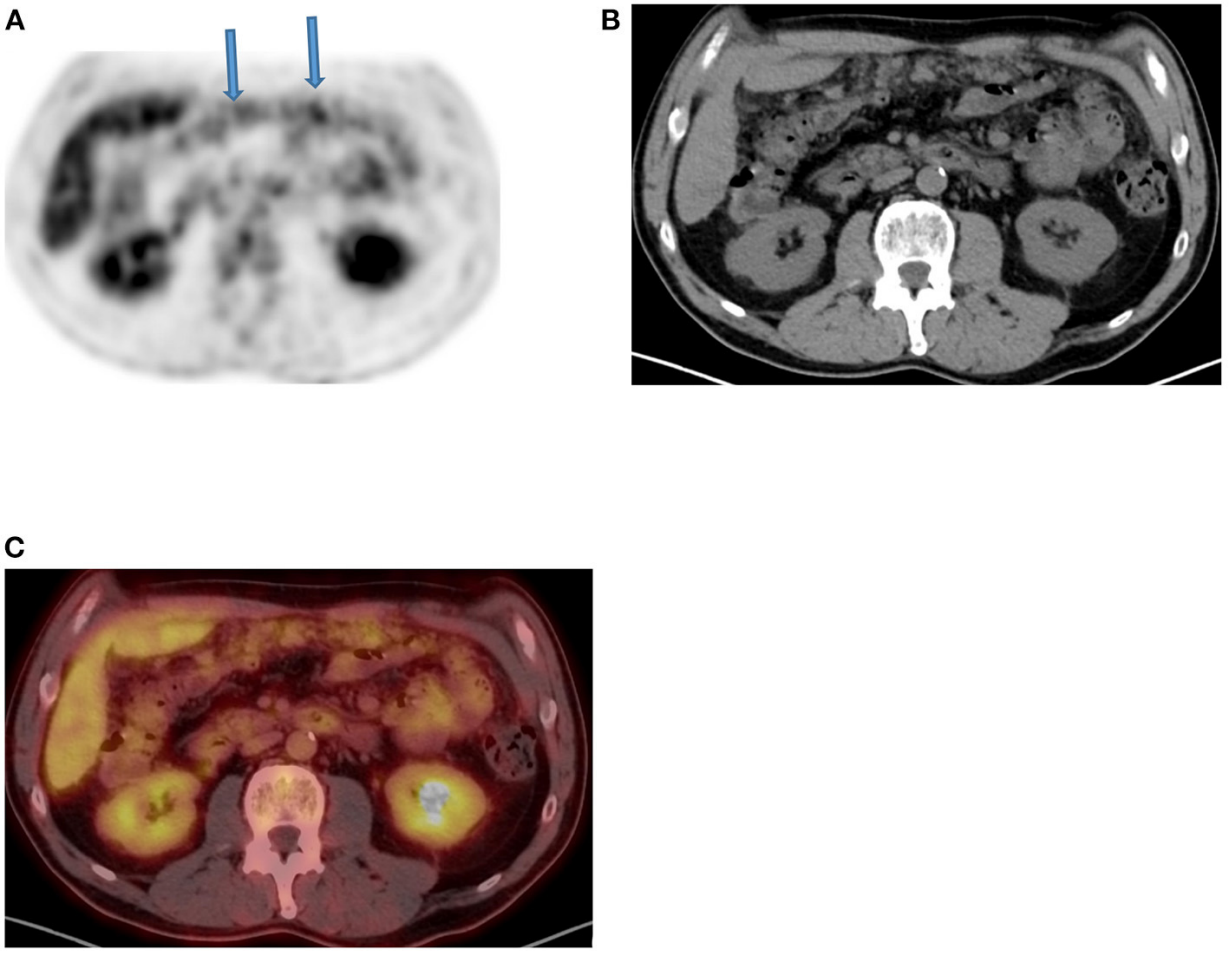
Omental cake. FDG PET Axial **(A)** image demonstrates diffuse moderate increased linear uptake in anterior abdomen in a “band like” patterns (arrows) that mimic large bowel uptake, in a patient with ovarian carcinoma who was referred for therapy response evaluation. Corresponding CT **(B)** and combined PET/CT **(C)** confirmed the features of “omental cake” that are suggestive of peritoneal metastasis.

The degree of FDG uptake alone may not be able to differentiate adenomas from colonic carcinomas. While several approaches are proposed on the use of SUVs to evaluate uptake, colonoscopy may be the standard to prevent missing malignancy (12–15). Regardless of the degree of uptake, focal bowel uptake is the most cause of high false positive rate since SUV values cannon distinguish between physiologic uptake and neoplasms (16). The higher rate of false positive findings is encountered in the cecum due to focal uptake in lymphoid tissue (17). In addition, inflammatory uptake can be seen in hemorrhoids. However, the patterns of FDG uptake may assist to differentiating potential causes. Local lesion is generally associated with nodular colonic uptake, segmental uptake is often associated with inflammation and diffuse uptake is usually normal (18). These described patterns should not be taken as cast in stones to avoid misinterpretation (Figure 11) and the CT component of PET/CT can also assist to identify certain entities with less doubt (Figure 12). Caution should be exercised if PET/CT is positive for recurrence in patients with normal CEA since most often follow up examinations do not show evidence of disease (3).

**Figure 13:**
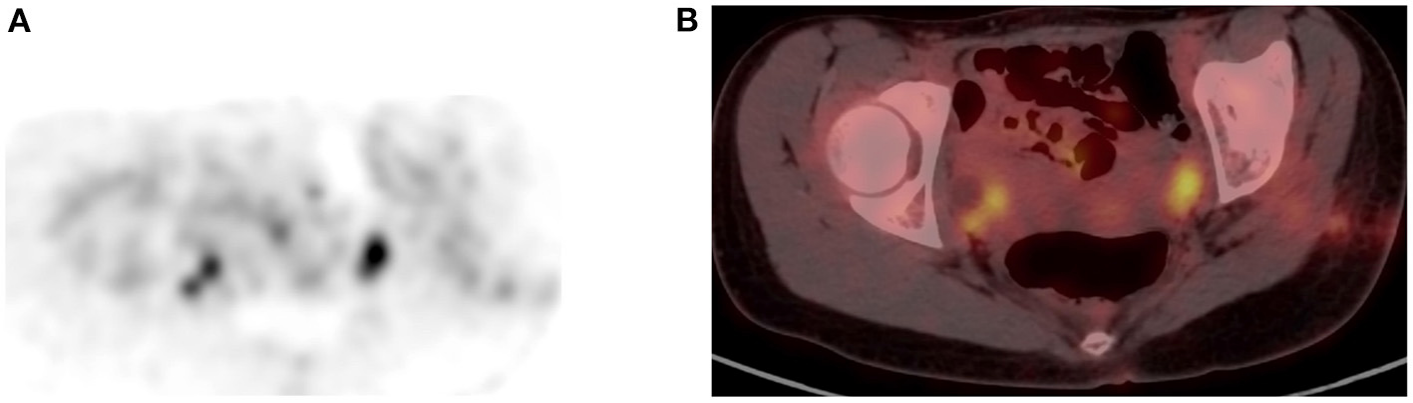
Uptake in ovaries. FDG PET Axial **(A)** image showed focal increased uptake in pelvis corresponding to ovaries in combined PET/CT **(B)** image, in a female patient of childbearing age. The uptake is physiologic due to menstrual cycle.

**Figure 14:**
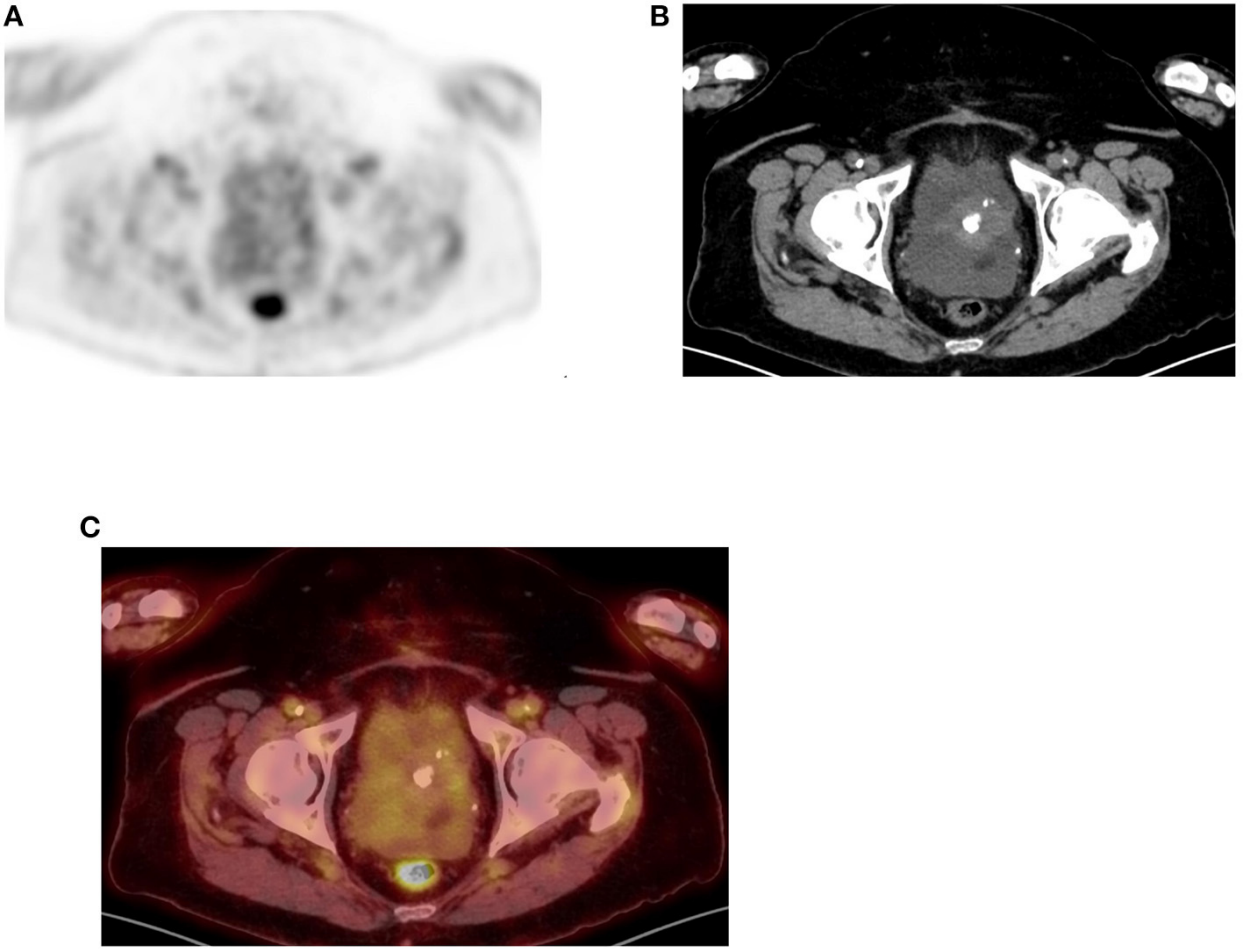
Uterine uptake. FDG PET Axial **(A)** image showing diffuse moderate uptake in central lower pelvis that correspond to calcified uterine fibroid as confirmed in CT and combined PET/CT **(B,C)**, in a 66-year-old female patient who was referred with non-alcoholic steatohepatitis (NASH) and ascites.

For gynecologic tumors, common false negative findings in patients with cervical cancer arise from small paraaortic lymph nodes (<0.5 cm) whereas isolated positive mediastinal nodes and will cause false positive results (19, 20). After treatment, the most common cause of false positive is a focal rectal activity. Mucinous and clear cell ovarian adenocarcinomas often do not show FDG uptake and cause false negative findings. Physiological ovarian uptake together with conditions like thecoma, endometriomas, benign cystic lesions as well as inflammatory processes are not uncommon and must be recognized. Increased FDG uptake related to ovulation as well as to early luteal phase of the menstruation is common in women of childbearing and may cause false positive results (1, 21). The latter may be difficult to be differentiated from pelvic nodal and focal bowel uptake (Figure 13). Similarly, FDG uptake may be seen in the endometrium in premenopausal women. The typical physiological endometrial uptake is diffuse whereas in cervical cancer, the FDG uptake is usually focal (21). In addition, FDG uptake is commonly seen in uterine fibroids (Figure 14) despite the benign nature (1).

**Figure 15:**
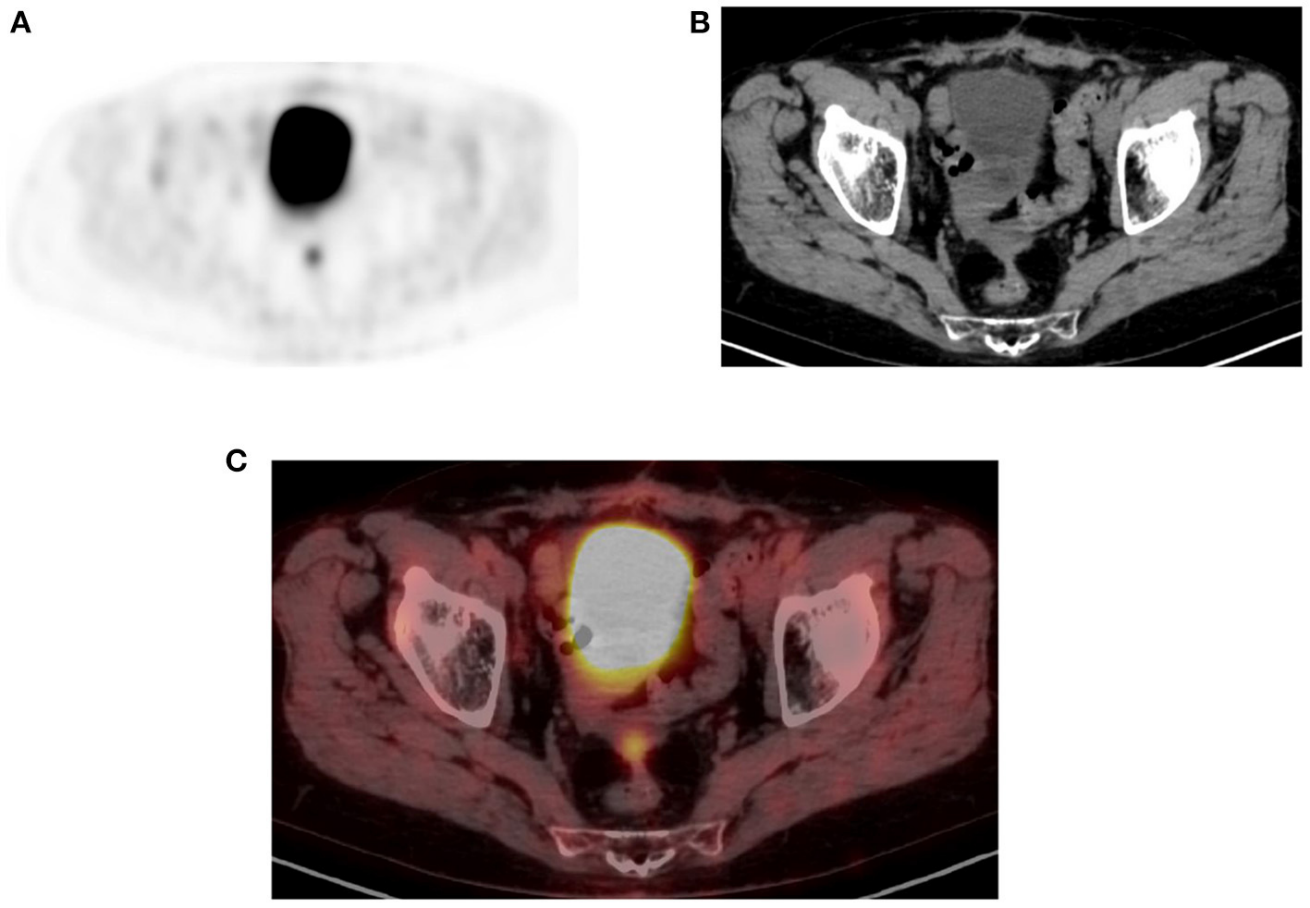
Physiologic pelvic focal uptake. FDG PET Axial **(A)** image showing focal increased uptake in posterior pelvic, in a 75-year-old female patient diagnosed with high grade serous carcinoma of the left ovary. She underwent oophorectomy and received chemotherapy. Her serum CA started to rise and was referred for restaging. The PET/CT showed active abdominal and pelvic adenopathy and in focal area of increased uptake that was localized in the vaginal fornix on CT **(B)** and combined PET/CT **(C)**.

In suspected active disease in the peritoneum, caution is required in windowing uptake in diffuse peritoneal carcinomatosis as it may not show focal lesions and be erroneously interpreted as negative findings (3). And not all focal uptake represents active disease (Figure 15).

**Figure 16:**
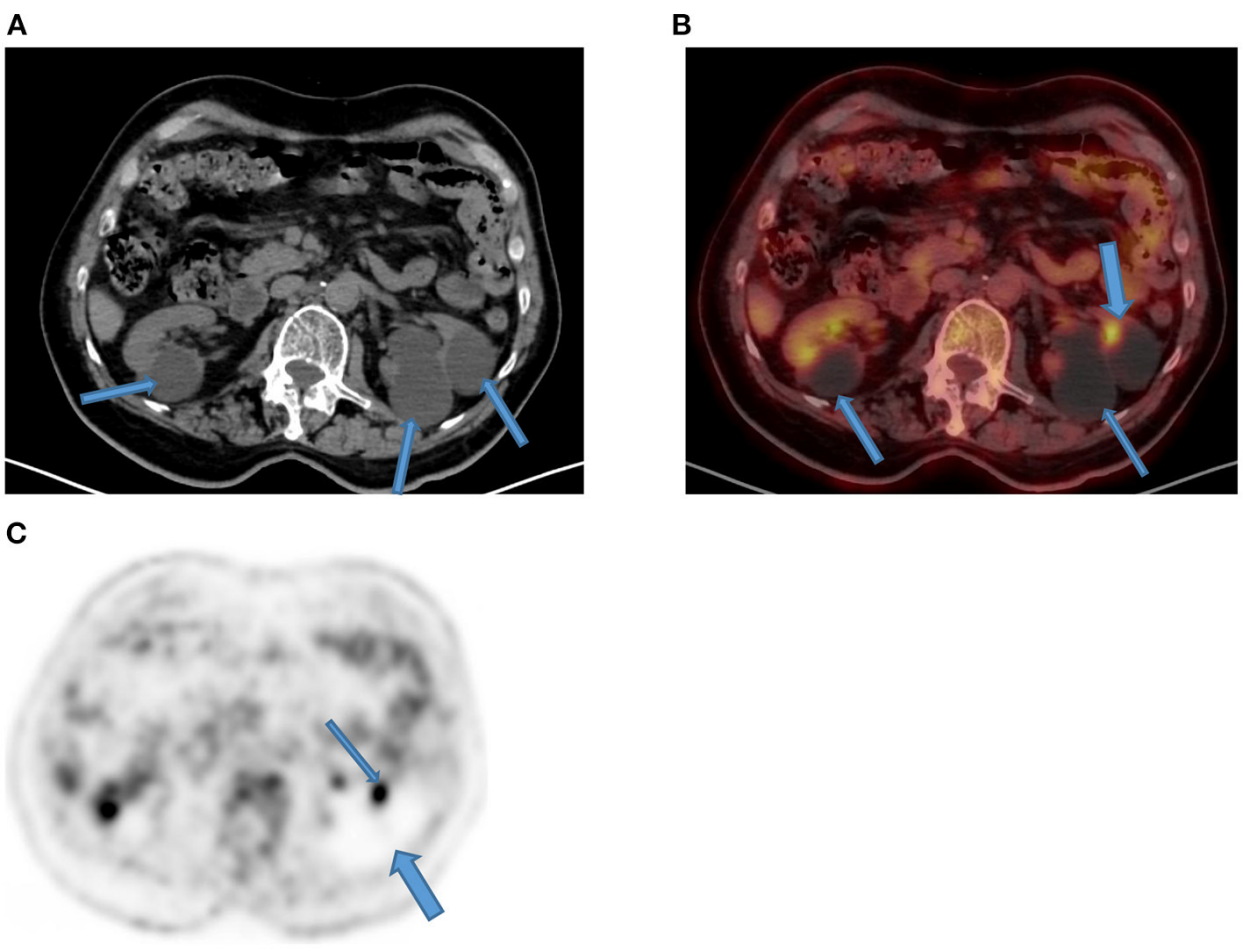
Focal kidney uptake. Axial CT **(A)** component of the PET/CT showed multiple bilateral Bosniak type 1 renal cysts that appear hypodense (arrows) as compared to thin normal renal tissue. Their magnitude in left kidney (thin arrows) showed compression and thinning of renal cortex with a focal FDG uptake (thick arrow) on corresponding combined PET/CT **(B)** that may mimic focal disease. Corresponding axial PET **(C)** demonstrates photon deficient areas due to renal cysts (thick arrow) with adjacent focal urinary uptake (thin arrow).

When imaging urological tumors, consider administrating diuresis in renal cell carcinoma if the study is done to assess the renal mass. Adjacent urinary activity may obscure the lesion thus causing a false negative result and at the same time focal FDG activity from urinary collection may mimic a lesion (Figure 16). Small (<1 cm) retroperitoneal lymph nodes are often missed on PET/CT (3).

**Figure 17:**
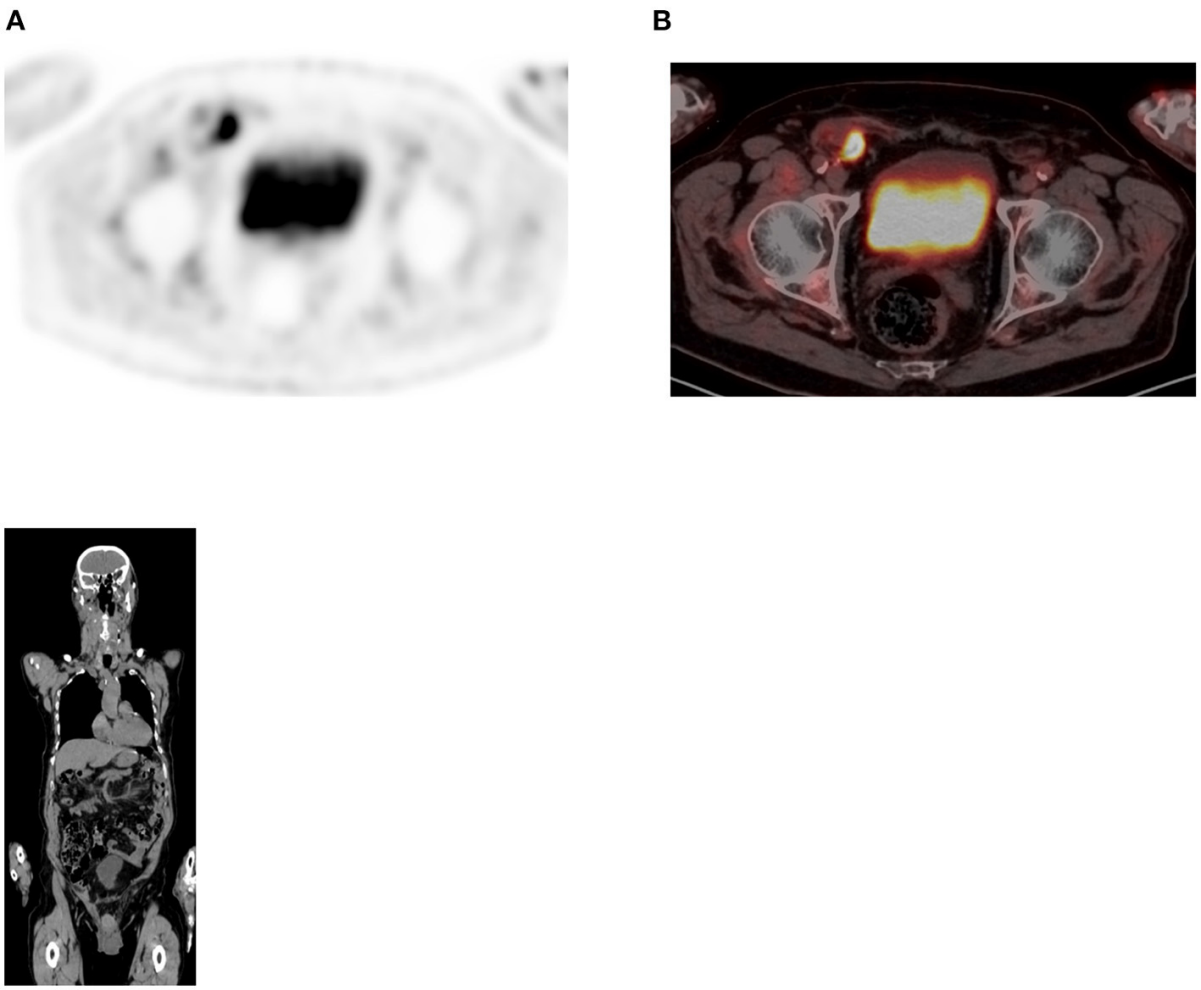
Uptake in hernia. FDG PET Axial **(A)** image showing focal increased uptake within right iliac fossa (RIF) in a 82 year old male patient with uncomplicated inguinal hernia as seen in combined PET/CT **(B)**. Coronal plane of the CT confirmed the small bowel content of the right inguinal hernia.

In patients of advanced aged, it is not uncommon to seen hernia, particularly inguinal types. Although complications remain rare, they may contain bowel with normal uptake that may mimic pathology (Figure 17). The same group age of patients, often urinary contamination may require careful correlation with CT for correct interpretation of the finding (1).

## Conclusion

PET/CT using 18F-FDG has revolutionized imaging of in oncology with direct impact on patients' management. This paper contributes toward minimizing interpreting mistake with its content and accompanying pictorial illustrations.

## Author Contributions

MV: conception, write up, and collection of images and review. JM: write up and collection of images. All authors contributed to the article and approved the submitted version.

## Conflict of Interest

The authors declare that the research was conducted in the absence of any commercial or financial relationships that could be construed as a potential conflict of interest.

## Publisher's Note

All claims expressed in this article are solely those of the authors and do not necessarily represent those of their affiliated organizations, or those of the publisher, the editors and the reviewers. Any product that may be evaluated in this article, or claim that may be made by its manufacturer, is not guaranteed or endorsed by the publisher.
